# Supplementary material for: Moving towards Integrated and Personalized Care in Parkinson’s Disease: A Framework Proposal for Training Parkinson Nurses
Source: J Pers Med. 2021 Jun 30;11(7):623. doi: 10.3390/jpm11070623 (PMC8304750; doi:10.3390/jpm11070623)
Supplement: Supplementary file 1 [file jpm-11-00623-s001.zip › jpm-1224535-supplementary.pdf]

Journal of Personalized Medicine

MDPI

SUPPLEMENTARY MATERIAL

Moving towards integrated and personalized care in Parkinson's disease: proposing a framework for training Parkinson Nurses

Table S1. Search Strategy and In- and Exclusion Criteria

Question	What are the care priorities for PD?
Inclusion	• Study Type:<br>o Clinical Study<br>o Quantitative or Qualitative Study<br>o Literature Review<br>o Conceptual or theoretical paper<br>o Clinical Guidelines<br>• Study Population:<br>o Patients with Parkinson´s Disease diagnosed as per United Kingdom Parkinson's Disease Society Brain Bank criteria and of either sex<br>• Setting: any setting<br>• Thematically:<br>o Describing an existing care model for Parkinson´s Disease<br>o Scientific guidelines regarding Parkinson´s Disease care<br>o Interventions implementing a care model for Parkinson´s Disease<br>o Theorizing care concepts or aspects of care<br>• Publication Date: no restriction<br>• Geographical location: no restriction<br>• Language: English or German
Exclusion	• Study type:<br>o Study Protocol<br>o Perspective/ Editorial<br>• Thematically:<br>o Models focusing on palliative care<br>o Systematic Literature Review not describing or theorizing a care model
Database:	MedLine; Web of Science
Search terms:	parkinson´s disease; concept; theory; model; care
Search Strategy (MedLine)	("parkinson disease"[MeSH Terms] OR ("parkinson"[All Fields] AND "disease"[All Fields]) OR "parkinson disease"[All Fields] OR ("parkinsons"[All Fields] AND "disease"[All Fields]) OR "parkinsons disease"[All Fields]) AND (("concept"[All Fields] OR "concept s"[All Fields] OR "concepts"[All Fields] OR ("theorie"[All Fields] OR "theories"[All Fields] OR "theory"[All Fields] OR "theory s"[All Fields]) OR ("model"[All Fields] OR "model s"[All Fields] OR "modeled"[All Fields] OR "modeler"[All Fields] OR "modeler s"[All Fields] OR "modelers"[All Fields] OR "modeling"[All Fields] OR "modelings"[All Fields] OR "modelization"[All Fields] OR "modelizations"[All Fields] OR "modelize"[All Fields] OR "modelized"[All Fields] OR "modelled"[All Fields] OR "modeller"[All Fields] OR "modellers"[All Fields] OR "modelling"[All Fields] OR "modellings"[All Fields] OR "models"[All Fields])) AND "care"[All Fields])
Search Strategy (Web of Science)	ALL=(parkinson disease OR (parkinson* AND disease) OR parkinson*) AND ALL=(concept* OR theorie* OR theory OR model*) AND ALL=(care*)

Table S2. Overview of Theoretical Concepts

Concept	Content
The Picker Institute Concept [64]	Patient-centeredness consists of:[1]<br>• Accessibility<br>• Information & communication<br>• Partner & family involvement<br>• Respect & autonomy<br>• Organization of healthcare<br>• Continuity of care<br>• Physical comfort<br>• Emotional support
A novel paradigm of continuous care in Parkinson´s Disease [73]	Care for PD should be enabled by three foundation pillars:<br>(i) Integrated care<br>(ii) Self-management support<br>(iii) Technology-enabled care
Active Aging Model for Parkinson's Disease [70]	Components of active aging, that are influenced by Parkinson´s Disease:<br>1. Physical activity<br>2. Mental well-being<br>3. Social communication<br>Potential consequences for:<br>-> Ability to learn over a lifetime<br>-> Employment status<br>-> Quality of life security<br>Modifying caring approaches: palliative care; self-care; patient-centered care & personalized care
A multi-element definition of continuity of care [72]	To experience continuity, care delivery services need:<br>• Continuity of information; continuity & coherence of medical record<br>• Cross-boundary and team continuity<br>• Flexible continuity<br>• Longitudinal continuity<br>• Relational or interpersonal continuity
Five A´s of self-management support [71]	Five A´s describe how the health-care professional can deliver SMS to the patient:<br>• Assessing beliefs, behaviors and knowledge<br>• Agreeing collaboratively on goals based on the patient's interest and confidence<br>• Advising the patient on health risks and benefits of change<br>• Arranging follow-ups<br>• Assisting the patient to identify personal barriers and develop problem-solving techniques
An Implementation Model for Integrated Care [74]	Variables for care integration:<br>• Type (i.e., organizational, professional, cultural, technological)<br>• Level (i.e., micro-, meso- and macro-)<br>• Process (management and organization of integrated care)<br>• Breadth (whole population vs. specific client group) degree/ intensity (informal linkages vs. fully integrated teams)<br><br>Key forms of integration: Horizontal, vertical, sectoral, people-centered, whole-system
The Knowledge-Translation Framework [80]	Eight steps for translating knowledge:<br>1. Identify a problem that needs addressing

	2. Identify, review, and select the knowledge or research relevant to the problem<br>3. Adapt the identified knowledge or research to the local context<br>4. Assess barriers to using the knowledge<br>5. Select, tailor, and implement interventions to promote the use of knowledge<br>6. Monitor knowledge use<br>7. Evaluate the outcomes of using the knowledge<br>8. Sustain ongoing knowledge use
The Web of Needs and Demands Model [65]	Living with PD means balancing within a web of needs and demands regarding:<br>1. Competence<br>2. Relatedness<br>3. Autonomy<br>-> A needs-based approach represents an important starting point to better understanding the consequences of living with PD
Five C´s for video-based therapy [28, 98]	Care; convenience; comfort; confidentiality & contagion
The Development Model of Integrated Care [66]	Nine-cluster model for quality management in integrated care:<br>• Patient-centeredness<br>• Delivery system<br>• Performance management<br>• Quality of care<br>• Result-focused learning<br>• Interprofessional teamwork<br>• Roles and tasks<br>• Commitment<br>• Transparent entrepreneurship<br><br>Organizational development happens across 4 phases were different priorities need to be addressed
World Health Organization responsiveness model [67]	Patient-centeredness consist of:[1]<br>• Confidentiality of information<br>• Communication<br>• Access to family and community support<br>• Autonomy<br>• Freedom to choose you own healthcare provider<br>• Dignity<br>• Prompt attention<br>• Quality of basic amenities
Oerem´s Self-Deficit Theory and the Nursing Process [68]	Nursing is needed when the client's therapeutic self-care demand exceeds the available self-care agency<br>The Nursing Process presents involves a method to determine self-care deficits and defining the roles of persons or nurses to meet the self-care demands:<br>1. Assessing (patients' health status; physicians and patients' perspective on the health status; goals within the context of life history, lifestyle, and health status; the person's requirements for self-care; the person's capacity to perform self-care)<br>2. Designing a care plan by organizing the patient's self-care demands and selecting a combination of helping methods<br>3. Implementation and evaluation

The Quality of Care Framework for Home-Based Medical Care [79]	Home-based care compromises categories of quality:<br>• Provider and practice activities<br>• Provider characteristics<br>• Outcomes for patients, caregiver and provider<br><br>➔ Can be assessed in 10 domains and 49 standards
Proactive and Integrated Management and Empowerment in Parkinson's Disease (PRIME) [75]	Proactive and Integrated Management and Empowerment in Parkinson's Disease has five main components:<br>1. Personalized care management<br>2. Education and empowerment of patients and carers<br>3. Empowerment of healthcare professionals<br>4. A population health approach<br>5. Support of the previous four components by patient- and professional-friendly technology
The Rainbow Model [76]	Care Services can be integrated across four domains:<br>1. Clinical<br>2. Professional<br>3. Organizational<br>4. Systems<br>And on different scales (individual <-> population) whereby functional and normative enablers play a role.<br>Ultimately, integration follows three aims: improving population health and experience of care while optimizing cost and utilization.
Six core elements of patient-centered care [27]	1. Emotional support, empathy and respect<br>2. Knowledge of PD treatment among caregivers<br>3. Provision of tailored information<br>4. Involvement in decisions and respect for patient preferences<br>5. Continuity and collaboration of caregivers<br>6. Accessibility of healthcare
Five Core Elements of Personalized Care Management for Parkinson´s Disease [78]	1. Information provision<br>2. Proactive monitoring of early detection signs and symptoms<br>3. Process monitoring<br>4. Care coordination<br>5. Patient navigation
The Chronic Care Model [77]	There are six elements, which can be addressed to improve chronic care:<br>• Organization of health care<br>• Design of the delivery system<br>• Decision support<br>• Clinical information system<br>• Self-management support<br>• Community resources<br>In order to deliver good self-management support, healthcare professionals should have knowledge about structures and available resources.

[1] adapted from [27]
